# Contraceptive failure with Copper T380A intrauterine device (IUD): A single tertiary center experience

**DOI:** 10.12669/pjms.325.10392

**Published:** 2016

**Authors:** Ali Ekiz, Burak Ozkose, Burak Yucel, Muhittin Eftal Avci, Ahmet Adanur, Gokhan Yildirim

**Affiliations:** 1 Ali Ekiz, MD. Department of Maternal-Fetal Medicine Unit, Kanuni Sultan Suleyman Training and Research Hospital, Istanbul, Turkey; 2 Burak Ozkose, Department of Obstetrics and Gynecology, Kanuni Sultan Suleyman Training and Research Hospital, Istanbul, Turkey; 3 Burak Yucel, Department of Obstetrics and Gynecology, Kanuni Sultan Suleyman Training and Research Hospital, Istanbul, Turkey; 4 Muhittin Eftal Avci, Department of Maternal-Fetal Medicine Unit, Kanuni Sultan Suleyman Training and Research Hospital, Istanbul, Turkey; 5 Ahmet Adanur, Department of Obstetrics and Gynecology, Kanuni Sultan Suleyman Training and Research Hospital, Istanbul, Turkey; 6 Gokhan Yildirim, Department of Maternal-Fetal Medicine Unit, Kanuni Sultan Suleyman Training and Research Hospital, Istanbul, Turkey

**Keywords:** Contraceptive failure, Copper, Intrauterine device, Pregnancy

## Abstract

**Objective:**

The objective of this study was to assess the risk factors of pregnancy with Copper (Cu)T380A IUD and pregnancy outcomes.

**Methods:**

A retrospective study evaluating the risk factors and pregnancy outcomes of 81 patients who conceived with CuT380A IUD *in situ*.

**Results:**

Four ectopic pregnancies and 77 intrauterine pregnancies were detected. Twenty-six pregnancies (33.76%, 26/77) were terminated according to maternal desire. Twenty-five patients (32.46%, 25/77) whose IUDs were removed constituted the Removed IUD Group, and the remaining 26 patients constituted IUD Left *in situ* Group. Term pregnancy rates (76% vs. 20.8%, p=0.002) were significantly higher in the Removed IUD Group compared with the IUD Left *in situ* Group. Abortion rates (16% vs. 53.84%, p=0.008) were detected significantly higher in the IUD Left *in situ* Group.

**Conclusion:**

The main result of our study was that pregnancy with CuT380A *in situ* is a significant risk factor for adverse perinatal outcome. Adjusting the scheduled follow-ups for checking the IUD seems to be important in order to prevent accidental pregnancy.

## INTRODUCTION

The intrauterine device (IUD) is the most widely used reversible method of contraception currently. The estimation is that 15% of the world’s women of reproductive age use it.^1^ The prevalence of users among countries is widely variable, from 1.8% in Oceania to 27.0% in Asia.^2^ Contraception with an intrauterine device is highly effective, long acting, and rapidly reversible after removal. Copper-containing IUDs continuously release a small amount of the metal and stimulate the formation of prostaglandins within the uterus. As a result, intrauterine devices cause the formation of “biologic foam” within the uterine cavity, which has a toxic effect on sperm and ova and impairs implantation.

Although IUDs are a highly effective contraceptive method, there are some factors limiting prefer ability, such as misinformation about the risks of ectopic pregnancy, infection and infertility.^3^ IUDs also have a wide spectrum of side effects; some are manageable and some of them are serious.

Intrauterine and especially ectopic pregnancies are important complications of intrauterine contraception. When a pregnancy occurs within a period of IUD usage, the first recommendation is to exclude the ectopic pregnancy. Unintended pregnancies as a contraceptive failure of IUDs are rare and their incidence is reported 1.4 per 100 at seven years for copper-releasing IUDs.^4^ The cumulative ectopic pregnancy rate was reported as 0.4% for Copper T380A (CuT380A).^5^ Some factors, especially displacement of device, play an important role in contraceptive failure.

Evaluation of risk factors of contraceptive failure and outcomes of the pregnancy that consist of contraceptive failure of CuT380A IUDs constitute the main objective of this study.

## METHODS

This retrospective study involved a thorough review of the patients who experienced accidental pregnancy with the CuT380A IUD between January 2013 and May 2015 in a tertiary center. In our Family Planning Unit (FPU), patients are given counselling about benefits, failure rates, risks and side effects of all contraceptive methods. When a patient selects a method, informed consent about selected contraceptive method is obtained from the patient. Currently available CuT380A IUD is licensed for 10 years duration. After completing mandatory evaluations including physical examination, medical history and sexually transmitted disease risk assessment, IUDS are inserted using appropriate method.

IUDs are inserted in three different periods, including the normal menstrual cycle, following abortion, or post-delivery period. We do not insert IUDs immediately after delivery. Although, an IUD may be inserted at any time during the menstrual cycle, all IUDs were inserted during the menstrual cycle after possible pregnancy is excluded. For women who were in the postpartum period, the insertions of IUDs were delayed at least 6 weeks after delivery. In some cases, IUDs were inserted into the patients immediately after first trimester abortions or elective pregnancy terminations. In Turkey, termination of pregnancy due to maternal desire is legally allowed until the 10^th^ week of gestation. All the patients who have IUD inserted are also recommended for scheduled follow-up visits at the end of first menses, 6 and 12 months after insertion, and yearly thereafter.

**Fig.1:**
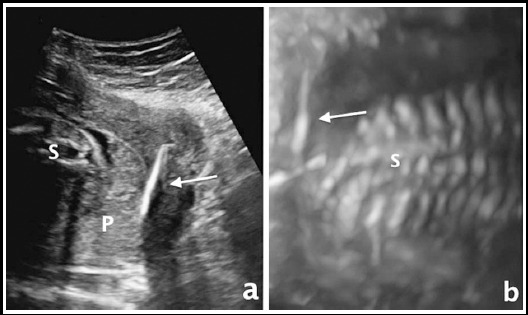
a) The embedded IUD between placenta and myometrium was shown via Gray Scale ultrasound. b) the closeness of IUD and spine of the fetus was shown by 3-dimentional ultrasound examinations with maximum (skeletal) mode. Both images were obtained from same patient who has 25 gestational weeks pregnancy with complicated by PPROM. Arrow: Intrauterine Device, S: Spine, P: Placenta, PPROM: Preterm premature rupture of membrane.

When pregnancy is detected in a patient with IUD, first evaluation is performed in order to exclude ectopic pregnancy. The exact location of pregnancies and IUDs are determined with ultrasonographic examination (Fig.1). The patients who were diagnosed with ectopic pregnancy with an IUD are managed according to physical examination, ultrasound and laboratory findings. Detailed counselling about risks and outcomes of intrauterine pregnancy with an IUD is given to patients who are diagnosed with intrauterine pregnancy. Each of the patients can prefer either termination of pregnancy or continue her pregnancy and thus, when a pregnant woman decides to continue her pregnancy, the IUD is removed if strings are visible.

The following findings constitute exclusion criteria: 1) patients who did not receive family planning counselling and had IUDs inserted at our FPU, 2) patients who choose IUD except CuT380A, 3) patients who were not followed up in our FPU and 4) patients who had incomplete medical records. The clinical data regarding patients’ features and type of IUD were achieved from our FPU records. The center`s institutional review board approved the study. Since the study was retrospective, informed consent was not obtained.

The statistical analysis was carried out using MedCalc (version 13.3, Mariakerke, Belgium) statistical software. Data are presented as mean ± standard deviation (SD). The Kolmogorov–Smirnov test assessed the normality of the distribution of continuous variables. A chi-squared test and Fisher’s exact test were used to analyse categorical variables, and a Student’s t-test was used for the analysis of normally distributed continuous variables. A Mann–Whitney U-test was used for non-normally distributed variables. P < 0.05 was deemed statistically significant.

## RESULTS

A total of 7,997 CuT380A IUDs were performed, with complete medical records, at our FPU from January 2013 to May 2015. During this 29-month period, the medical records of patients who experienced pregnancy due to contraceptive failure of CuT380A were evaluated. After exclusion of other types of IUDs, in total, 81 patients were included from the records. We enrolled 81 pregnancies with CuT380A IUD *in situ* to the study. The incidence of contraceptive failure of CuT380A IUD was calculated as 1.01% (81/7997).

Four patients were diagnosed with ectopic pregnancy and all of them were treated with laparoscopic surgery. All pregnancies were detected at ampulla of the tube. The remaining 77 pregnancies were evaluated in three groups. Initially, Twenty-six patients (33.76%, 26/77) who desired termination of unwilling pregnancy constituted the Termination Group. Secondly, Twenty-five patients (32.46%, 25/77) whose strings of IUDs are visible constituted the Removed IUD Group and the finally 26 patients (33.76%) constituted the IUD Left *in situ* Group. Demographic characteristics and pregnancy details of the patients are shown in Table-I.

**Table-I:** Demographic characteristics and pregnancy details of the patients.

	*Mean ± SD*	*Range*
Age	30,68±6,3	19 – 42
Gravida	3,47±1,2	2 – 8
Para	2,30±1,0	1 – 5
Curettage	0,21±0,4	0 – 2
VB	1,55±1,4	0 – 5
Caesarean	0,58±0,9	0 – 4
Diagnosis of Pregnancy (Weeks)	6,74±2,4	4 – 16
Time of Abortion (Weeks)	11,80±4,7	8 - 25
Duration of IUD (years) at pregnancy detection	2,74±2,4	1 - 13

VB: Vaginal birth, IUD: Intrauterine device.

In the Removed IUD Group, 19 (76%, 19/25) term deliveries were observed; four pregnancies were complicated with abortion and two were complicated with preterm delivery. In the IUD Left *in situ* Group, 8 (20.8%, 8/26) term deliveries were observed; 18 pregnancies exhibited some complications, including abortion (53.84%, 14/26), preterm premature rupture of membrane (PPROM) (7.69%, 2/26), preterm delivery (3.84%, 1/26) and stillbirth (3.84%, 1/26). Among the abortions two of them were septic abortions in Left *in situ* group. Term pregnancy rates (76% vs. 20.8%) were significantly higher in the Removed IUD Group, compared with the IUD Left *in situ* Group (p = 0.002, chi square test). Abortion rates (16% vs. 53.84%) were detected significantly higher in IUD Left *in situ* Group (p = 0.008). Distribution of the complications of both groups is shown in Table-II.

**Table-II:** The outcome of pregnancy with IUD.

*Pregnancy Outcome*	*IUD Removed (n=25)*	*Pregnancy with IUD (n=26)*	p*
Term Delivery	19 (76%)	8 (32 %)	0.002
Preterm Delivery	2 (8 %)	1 (4 %)	0.61
Abortion	4 (16 %)	14 (56 %)	0.008
PPROM	0	2 (8 %)	0.49
Stillbirth	0	1 (4 %)	1.0

PPROM: Preterm Premature Rupture of Membrane,

* Chi Square test

**Fig.2:**
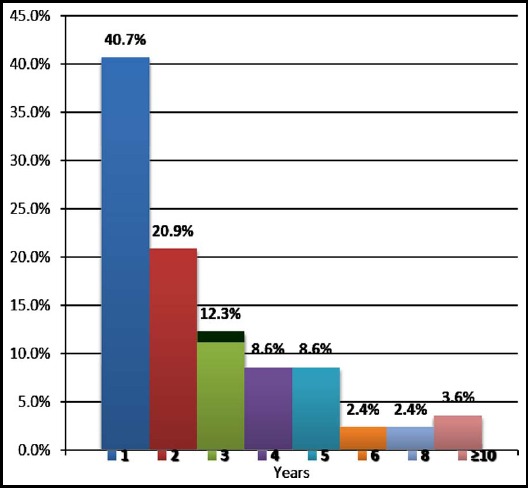
Distribution of years of IUD usage in which pregnancy detection.

The distribution of years, which defines the duration of IUD usage at time of pregnancy detection, is shown in Fig-2. According to the table, contraceptive failure rate is most frequent within the first year of insertion, with 40.7% among the pregnancies with IUDs.

As expected, the patients who were not adjusted to scheduled follow-ups that were advised at the time of contraceptive counselling constituted 61% of all patients. Additionally, the ratio of nursing mothers at the time of IUD insertion among all insertions was lower when compared with the Pregnancy with IUD Group. When analyzed, statistically significant difference between the ratios (37.97% versus 51.85%) was found via chi square test (p = 0.0097).

## DISCUSSION

Despite the high percentage of effectiveness noted so far, pregnancy in the presence of an IUD can occur, and the incidence is reported between 0.5 and 0.8 per 100^6^,^7^ at first year and cumulative pregnancy rates were 1.4 – 1.6 at seven years for the copper-releasing IUDs.^4^,^8^ Ectopic pregnancy is one of the most reported complications of contraceptive failure with an IUD.^4^,^9^ Pregnancy with an IUD *in situ* has a greater risk for adverse pregnancy outcomes, including miscarriage, septic abortion, chorioamnionitis, and preterm delivery compared with the general obstetric population.^10^

The risk of pregnancy with IUD was reported highest in the first year of insertion.^5^ According to our results, 40.7% of pregnancies occurred within the first year of IUD insertion. Additionally, second- and third-year pregnancies constituted 20.9% and 12.3% of pregnancies among the study population. Our results revealed that first-year and cumulative pregnancy rates with CuT380A (0.41% and 1.01% respectively) are compatible with the literature.

The World Health Organization published some recommendations upon the event of a pregnancy in the presence of an IUD.^11^ The recommendations include firstly ruling out possible ectopic pregnancy and secondly removing the IUD if strings are visible. It is generally agreed that removing of the IUD if strings are visible reduces the likelihood of obstetric complications.^12^,^13^ Although Schiesser et al. reported high success rate with few complications in ultrasound-guided extraction of IUD in patients with non-visible strings during cervical examination, it needs to be studied further.^14^

Several studies in the medical literature have investigated the associations between adverse perinatal outcome and pregnancy with a CuT380A IUD. Recently published review by Brahmi D. et al. demonstrated that pregnancies with an IUD *in situ* are at risk for adverse pregnancy outcomes, including spontaneous abortion, preterm delivery, septic abortion and chorioamnionitis.^10^ Additionally, in the same study, the authors noted that Cu-IUD removal decreased risks but not to the baseline risk of pregnancies without an IUD. In a recently published study by Ozgu-Erdinc SA et al., the combined risk of adverse pregnancy outcomes (miscarriage, intrauterine fetal death, intrauterine growth retardation, preterm birth and preterm premature rupture of membranes) was reported significantly low (36.8%) in the Removed IUD Group when compared with the Retained IUD Group (63.3%).^15^ Compatible with the current literature, our results demonstrated that term pregnancy rates without any obstetrical complication were significantly higher in the Removed IUD Group when compared with IUD Left *in situ* Group.

On the other hand, there might be some risk factors that play a role in contraceptive failure; dislocation of device is best known. Moreover, dislocation of device was reported to be an important factor affecting the course of pregnancy, a phenomenon most common in the first year of IUD usage.^11^,^16^ Among the study population, the patients who were not adjusted to scheduled follow-ups constituted 61% of all patients. Based on these findings, it is important to advise to all IUD users to adjust scheduled follow-ups after insertion of IUD.

Although breastfeeding provides protection against pregnancy via prolongation of anovulation, in our study, the ratio of nursing mothers at the time of IUD insertion were significantly higher in Pregnancy with IUD Group compared with all insertions, which means that there may be some factors that play a role in dislocation of IUD; perhaps the factor is increasing uterine contractility by breastfeeding. For instance, Oxytocin secretion, which is stimulated by breastfeeding may contribute to dislocation of device. On the other hand, another factor such as maternal neglect during the postpartum period may cause adapting failure to follow-ups of IUDs. Of course the difference can cause relatively limited number of pregnancies, which evaluated in the current study and, nevertheless, a question being raised about whether breastfeeding has an effect on contraceptive failure with IUD. This question will require further well-designed prospective studies.

This study was performed in a single tertiary center and only CuT380A IUD users were evaluated. The complicated pregnancies were compared according to IUD removal or left in situ. The distributions of the duration of IUD usage at time of pregnancy detection are highlighted in detail. All these important points constituted the strengths of the study. On the other hand, being a retrospective study and relatively small sample size of evaluated pregnancies constituted the limitation of the study.

According to our results, 33.7% of cases among the Turkish population decided to undergo termination of unwilling pregnancy because of socioeconomic and/or psychological reasons. The main result of our study was that pregnancy with CuT380A *in situ* is a significant risk factor for adverse perinatal outcome. Adjusting the scheduled follow-ups for checking the IUD seems to be important in preventing accidental pregnancy. The associations between breastfeeding and contraceptive failure with IUD may be worth to investigate with further well-designed studies.
